# Treatment of uterine prolapse stage 2 or higher: a randomized multicenter trial comparing sacrospinous fixation with vaginal hysterectomy (SAVE U trial)

**DOI:** 10.1186/1472-6874-11-4

**Published:** 2011-02-15

**Authors:** Renée J Detollenaere, Jan den Boon, Jelle Stekelenburg, Akeel HH Alhafidh, Robert A Hakvoort, Mark E Vierhout, Hugo WF van Eijndhoven

**Affiliations:** 1 Dept. of Obstetrics and Gynaecology, Isala klinieken Zwolle, the Netherlands; 2 Dept. of Obstetrics and Gynaecology, Medisch Centrum Leeuwarden, the Netherlands; 3 Dept. of Obstetrics and Gynaecology, Ziekenhuis Groep Twente Hengelo, the Netherlands; 4 Dept. of Obstetrics and Gynaecology, Spaarne ziekenhuis Hoofddorp, the Netherlands; 5 Dept. of Obstetrics and Gynaecology, Radboud University Nijmegen Medical Centre, Nijmegen, the Netherlands

## Abstract

**Background:**

Pelvic organ prolapse is a common health problem, affecting up to 40% of parous women over 50 years old, with significant negative influence on quality of life. Vaginal hysterectomy is currently the leading treatment method for patients with symptomatic uterine prolapse. Several studies have shown that sacrospinous fixation in case of uterine prolapse is a safe and effective alternative to vaginal hysterectomy. However, no large randomized trials with long-term follow-up have been performed to compare efficacy and quality of life between both techniques.

The SAVE U trial is designed to compare sacrospinous fixation with vaginal hysterectomy in the treatment of uterine prolapse stage 2 or higher in terms of prolapse recurrence, quality of life, complications, hospital stay, post-operative recovery and sexual functioning.

**Methods/design:**

The SAVE U trial is a randomized controlled multi-center non-inferiority trial. The study compares sacrospinous fixation with vaginal hysterectomy in women with uterine prolapse stage 2 or higher. The primary outcome measure is recurrence of uterine prolapse defined as: uterine descent stage 2 or more assessed by pelvic organ prolapse quantification examination and prolapse complaints and/or redo surgery at 12 months follow-up. Secondary outcomes are subjective improvement in quality of life measured by generic (Short Form 36 and Euroqol 5D) and disease-specific (Urogenital Distress Inventory, Defecatory Distress Inventory and Incontinence Impact Questionnaire) quality of life instruments, complications following surgery, hospital stay, post-operative recovery and sexual functioning (Pelvic Organ Prolapse/Urinary Incontinence Sexual Questionnaire). Analysis will be performed according to the intention to treat principle. Based on comparable recurrence rates of 3% and considering an upper-limit of 7% to be non-inferior (beta 0.2 and one sided alpha 0.025), 104 patients are needed per group.

**Discussion:**

The SAVE U trial is a randomized multicenter trial that will provide evidence whether the efficacy of sacrospinous fixation is similar to vaginal hysterectomy in women with uterine prolapse stage 2 or higher.

**Trial registration:**

Netherlands Trial Register (NTR): NTR1866

## Background

Pelvic organ prolapse (POP) is a common health problem affecting up to 40% of parous women over 50 years old [1]. The life-time risk for women to undergo surgery for the management of POP is about 11% and 30% of these women will need additional surgery because of prolapse recurrence [2]. The risk of POP increases with the number of vaginal births and is higher in older and obese women. POP has significant negative effects on a woman's quality of life, ranging from physical discomfort, psychological and sexual complaints to occupational and social limitations.

POP is defined as the descent of one or more of the pelvic organs. Anterior vaginal wall prolapse concerns the bladder and/or urethra (cystocele, urethrocele). Apical prolapse entails either the uterus or post-hysterectomy vaginal cuff. Posterior vaginal wall prolapse concerns the rectum but can also include the small or large bowel (rectocele, enterocele). Women can present with prolapse of one or more compartments. We will focus on the treatment of uterine prolapse in this study.

In the Netherlands vaginal hysterectomy is currently the leading treatment method for patients with symptomatic uterine prolapse. Although the literature is inconclusive, it has been suggested that hysterectomy may cause nerve supply damage and disrupt supportive structures of the pelvic floor. Therefore women may be at increased risk for bladder dysfunction and new-onset stress incontinence after vaginal hysterectomy [3-5]. The incidence of post-hysterectomy vaginal vault prolapse varies between 0,2 and 12% [6-8]. Hysterectomy for pelvic organ prolapse appears to be a particular risk factor. The risk of prolapse repair after hysterectomy was 4.7 times higher in women whose initial hysterectomy was indicated for pelvic organ prolapse and 8 times higher if preoperative prolapse grade 2 or more was present [9].

In several retrospective and prospective studies it has been shown that sacrospinous fixation in case of uterine or vaginal vault prolapse is a safe and effective treatment [10-14]. Two sutures suspend the cervix or vaginal vault to the sacrospinous ligament bringing the apex above the levator plate. The procedure is associated with a few serious complications. Buttock pain on the side where the sacrospinous sutures have been passed occurs in approximately 10-15% of the women but typically resolves in days to months.

Two retrospective and one prospective study comparing vaginal hysterectomy to sacrospinous fixation demonstrated no significant difference in anatomical outcome, while hospital stay was shorter, less pain was experienced and recovery was quicker in the latest group [15-17]. However to date only one randomised study comparing both procedures is available. This multi-center trial compared vaginal hysterectomy to sacrospinous fixation in a group of 66 women with uterine descent and found a higher rate of recurrences after one year in patients with sacrospinous fixation (27% versus 3% recurrence in patients with vaginal hysterectomy) [18]. This conflicting evidence could be attributed to inadequate statistical power owing to small sample size and short duration of follow up. Possible other explanations for the difference in recurrences rates between the different studies are heterogeneity of data collection and selection bias, for instance excluding women with a stage 4 uterine descent. Also due to the multi-center design of the study more gynaecologists performed the procedures, possibly by using different techniques, and therefore introducing the risk of a difference in quality.

Benefits from sacrospinous fixation described in previous studies were also demonstrated in the randomised trial. Median hospital stay was shorter after sacrospinous fixation (3 versus 4 days) and patients had earlier resumption of daily activities and work (43 days versus 66 days).

In conclusion, studies that compare vaginal hysterectomy to sacrospinous fixation lack long-term follow-up and have insufficient power because of the small numbers and heterogeneity of included patients. Therefore we will conduct a multi-centre, non-inferiority trial to determine whether the efficacy of sacrospinous fixation is similar to vaginal hysterectomy in women with symptomatic uterine prolapse pelvic organ prolapse quantification (POP-Q) stage 2 or higher.

## Methods/design

### Study objectives

The objective of this study is to compare sacrospinous fixation with vaginal hysterectomy in the treatment of uterine prolapse POP-Q stage 2 or higher in terms of recurrence of prolapse, quality of life, complications, post-operative recovery, hospital stay and sexual functioning.

#### Hypothesis

Our hypothesis for this study is that there is no difference in recurrence rate between sacrospinous fixation and vaginal hysterectomy in symptomatic women with uterine decent POP-Q stage 2 or higher. However, sacrospinous fixation may be associated with shorter hospital stay, more quick recovery and less postoperative pain.

### Study design

**Figure 1:**
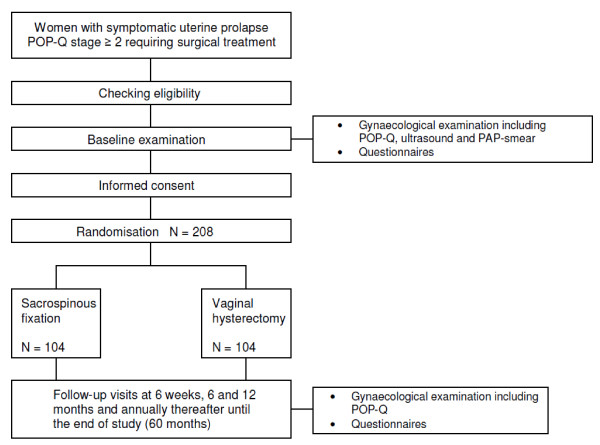
**Study design**.

The SAVE U trial is a prospective randomized non-blinded clinical trial conducted with the aim to determine non-inferiority of the primary endpoint between sacrospinous fixation and vaginal hysterectomy. The study will be an open label study, as it is impossible to blind the health care workers and patients involved for the surgical procedure to which the woman is allocated. Follow-up after one year, however, will be done by an independent physician. After inclusion, patients will be randomized centrally in 1:1 ratio stratified per centre and severity of prolapse. Patients are followed-up at 6 weeks, 6 months, 12 months and annually thereafter till 60 months follow-up. The design is presented in figure 1.

### Study population and recruitment

All women seeking treatment for symptomatic pelvic organ prolapse with uterine descent POP-Q stage 2 or higher, will be considered for inclusion in the SAVE U trial. Patients with co-existing anterior/posterior defects or concomitant incontinence surgery can be included.

Women with previous pelvic floor or prolapse surgery, known malignancy or abnormal cervical smears, a wish to preserve fertility, language barriers, presence of immunological/haematological disorders interfering with recovery after surgery, abnormal ultrasound findings of uterus or ovaries or abnormal uterine bleeding and who are unwilling to return for follow-up are excluded from the study.

Assessment for eligibility is performed by gynaecologist and/or residents of the participating hospital. Patients eligible for participation are counselled about the long duration of follow up that is involved in the study. Also the risks associated with uterus preservation is clearly outlined. Subsequently, written patient information is provided. An interval of one to two weeks between the primary visit and the next appointment allows sufficient time for women to think about participation. Written informed consent is obtained before randomisation.

### Primary and secondary endpoints

The primary endpoint of this study is surgical failure, defined as recurrence of prolapse POP-Q stage 2 of the middle compartment and prolapse complaints and/or redo surgery. This item will be evaluated by performing a POP-Q examination at 12 months follow-up.

Secondary endpoints of this study include: subjective outcome and improvement in general and disease-specific quality of life, complications, hospital stay, post-operative recovery and sexual functioning after sacrospinous fixation or vaginal hysterectomy.

### Participating hospitals

Four Dutch (non-academic) hospitals will enrol patients.

### Randomisation

After patients have consented for participation in the study, they are randomized centrally through a website using computer-generated randomisation tables. The subjects are assigned in a 1:1 ratio to either sacrospinous fixation or vaginal hysterectomy. Randomisation will be stratified according to centre and severity of prolapse (POP-Q stage 2, 3 or 4). The details of the series are unknown to investigators or to the participating gynaecologists and all participants will receive unique study numbers.

### Data collection

All patients will undergo routine gynaecological examination which is part of standard procedure before surgery. This includes pelvic ultrasound to exclude uterine or ovarian disease, routine PAP-smear and vaginal inspection in 45° semi-upright position for staging uterovaginal prolapse by a POP-Q examination. The POP-Q system has been developed by the international Continence Society and is a reliable and specific method to measure organ support [19,20]. Maximum prolapse is demonstrated and identified by asking the patient to cough and to perform a Valsava manoeuvre while each vaginal wall is individually exposed.

At inclusion all patients are requested to fill in validated quality of life questionnaires (RAND 36, Euroqol 5D, Urogenital Distress Inventory, Defecatory Distress Inventory, Incontinence Impact Questionnaire) [21-26] and two questionnaires regarding sexual functioning (Pelvic Organ Prolapse/Urinary Incontinence Sexual Questionnaire and selected items from the 'Vragenlijst Seksuele disfuncties') [27-29]. Preoperative urodynamic evaluation is only performed in women with bladder dysfunction. During hospitalisation and the first 6 weeks after surgery patients keep a diary which contains the following items: post-operative pain measured by the Visual Analogue Score (VAS), used pain medication and the Recovery Index-10 (RI-10) which is a validated quality-of life questionnaire measuring subjective postoperative recovery [30]. After surgery patients will visit the hospital at 6 weeks (routine post-operative consultation), 6 months, 12 months and yearly thereafter till 60 months follow-up.

### Interventions

Eligible women will be randomly allocated to receive either a sacrospinous fixation or a vaginal hysterectomy. All procedures will be performed under general anesthesia or spinal analgesia according to the preference of patient and anesthesiologist. All women receive peri-operative antibiotics and thrombosis prophylaxis. Post-operatively a bladder catheter is placed and removed according to local hospital protocol. Patients will receive analgesics if necessary in accordance with local hospital protocol. All patients are advised to abstain from heavy physical work for a minimal period of 6 weeks.

#### Sacrospinous fixation

At least twenty procedures must have been performed by participating gynaecologists to eliminate a learning curve effect. All procedures are performed unilaterally to the right sacrospinous ligament. Access to sacrospinous ligament is obtained through the pararectal space. The posterior vaginal wall will be incised and separated from the rectum. The right ischial spine will be localised digitally and after retractor positioning the ligament is made visible through blunt dissection. Two permanent sutures (Prolene 1.0, Ethicon, Somerville, NJ, USA) will be placed through the right sacrospinous ligament at least 2 cm from the ischial spine. Hereafter, an additional anterior and/or posterior colporrhaphy or incontinence surgery can be performed. The permanent sutures will be placed through the posterior side of the cervix and two thirds of the posterior vaginal wall will be closed with absorbable sutures (Vicryl 2, Ethicon, Somerville, NJ, USA). The permanent sutures will be tightened and the cervix redressed. The remainder of the vaginal wall will be closed.

#### Vaginal hysterectomy

The patient is placed in lithotomy position and a tenaculum forceps is used to grasp the cervix. The vaginal wall around the cervix is circumcised. The bladder will be dissected and the anterior peritoneum opened. The posterior peritoneum will be opened and the Douglas cul-de-sac is entered. The uterosacral ligaments will be identified, transected and ligated. In several steps the uterus will be removed using clamps and sutures. Following removal of the uterus, the adnexa are inspected and the surgical pedicles are inspected for bleeding. The peritoneum is closed in a purse-string manner using a delayed-absorbable suture (Vicryl 1.0). The ligature of the uterosacral ligaments is sutured to the vaginal cuff to aid in long-term vaginal support. The vaginal wall incision is closed left to right with interrupted sutures.

During the same procedure additional anterior and/or posterior colporrhaphy or incontinence surgery can be performed.

### Statistical analysis

#### Sample size and power considerations

The sample size for this trial has been estimated using the hypothesis that both interventions are equivalent regarding anatomical outcome. The aim is to show that in the sacrospinous fixation arm, anatomical result is comparable to the vaginal hysterectomy group. Two groups of 94 patients will be included to yield a 80% power for a non-inferiority margin of 7%, assuming a relapse rate of 3% [18]. Considering a 10% loss in follow-up 104 women per arm are needed and thus a total of 208 women.

#### Data analysis

Patient characteristics will be summarized using descriptive statistics for continuous variables presented with medians, means and standard deviations as appropriate. Categorical data will be presented as rates and percentages. Anatomical outcome and recurrence rate assessed by a POP-Q-examination in both study groups will be considered as primary outcome. Surgical failure (recurrence) is defined as the presence of prolapse stage 2 or more in the middle compartment with prolapse complaints and/or redo surgery at one year follow-up. Non-inferiority of sacrospinous fixation to vaginal hysterectomy will be concluded if the lower limit of the 95% confidence interval lies above the non-inferiority margin of -7% (this is equivalent to performing a one-sided hypothesis test at the 0.025 level of significance, based on the null hypothesis that sacrospinous fixation is inferior to vaginal hysterectomy). If the 95% confidence interval for the difference in recurrence rates not only lies above the non-inferiority margin, but also above zero then it will be concluded that there is evidence of superiority of sacrospinous fixation over vaginal hysterectomy in terms of statistical significance at the 2-sided 5% level (p < 0.05).

### Ethics

The study is conducted in accordance with the principles of the Declaration of Helsinki and 'good clinical practice' guidelines. The SAVE U trial has been approved by the Medical Ethical Committee of the Isala Klinieken Zwolle (MEC 09/625) and the local Ethical Committees of the participating centers. Prior to randomization informed consent will be obtained.

## Discussion

This is a protocol for a randomised trial comparing sacrospinous fixation and vaginal hysterectomy for the treatment of uterine prolapse POP-Q stage 2 or higher with regard to anatomical outcome, post-operative recovery, length of hospital stay, complications and sexual functioning.

The findings of this trial will contribute to answer the question which surgical treatment is preferable in women with symptomatic uterine prolapse POP-Q stage 2 or higher. If equivalence in anatomical outcome is found, the comparison of the secondary outcomes will be essential in selecting the preferred strategy.

## Competing interests

The authors declare that they do not have competing interests.

## Authors' contributions

RJD, JB and HWFE contributed to the development of the trial protocol. RJD drafted this manuscript and has responsibility for the logistical aspects of the trial. All authors co-authored the manuscript and approved the final version.

## Pre-publication history

The pre-publication history for this paper can be accessed here:

http://www.biomedcentral.com/1472-6874/11/4/prepub

## References

[B1] Slieker-ten Hove MC Pool-Goudzwaard AL Eijkemans MJ Steegers-Theunissen RP Burger CW Vierhout ME The prevalence of pelvic organ prolapse symptoms and signs and their relation with bladder and bowel disorders in a general female population Int Urogynecol J Pelvic Floor Dysfunct 2009 20 1037 45 10.1007/s00192-009-0902-1 19444368 PMC2721135

[B2] Olsen AL Smith VJ Bergstrom JO Colling JC Clark AL Epidemiology of surgically managed pelvic organ prolapse and urinary incontinence Obstet Gynecol 1997 89 501 6 10.1016/S0029-7844(97)00058-6 9083302

[B3] Altman D Granath F Cnattingius S Falconer C Hysterectomy and risk of stress-urinary-incontinence surgery: nationwide cohort study Lancet 2007 370 1494 9 10.1016/S0140-6736(07)61635-3 17964350

[B4] Mant J Painter R Vessey M Epidemiology of genital prolapse: observations from the Oxford Family Planning Association Study Br J Obstet Gynaecol 1997 104 579 85 10.1111/j.1471-0528.1997.tb11536.x 9166201

[B5] Blandon RE Bharucha AE Melton LJ Schleck CD Babalola EO Zinsmeister AR Gebhart JB Incidence of pelvic floor repair after hysterectomy: A population-based cohort study Am J Obstet Gynecol 2007 197 664 10.1016/j.ajog.2007.08.064 18060973 PMC2562278

[B6] Marchionni M Bracco GL Checcucci V Carabaneanu A Coccia EM Mecacci F Scarselli G True incidence of vaginal vault prolapse. Thirteen years of experience J Reprod Med 1999 44 679 684 10483537

[B7] Barrington JW Edwards G Posthysterectomy vault prolapse Int Urogynecol J Pelvic Floor Dysfunct 2000 11 241 245 10.1007/s001920070033 11005477

[B8] Dällenbach P Kaelin-Gambirasio I Jacob S Dubuisson JB Boulvain M Incidence rate and risk factors for vaginal vault prolapse repair after hysterectomy Int Urogynecol J Pelvic Floor Dysfunct 2008 19 1623 9 18773134 10.1007/s00192-008-0718-4

[B9] Dällenbach P Kaelin-Gambirasio I Dubuisson JB Boulvain M Risk factors for pelvic organ prolapse repair after hysterectomy Obstet Gynecol 2007 110 625 32 17766610 10.1097/01.AOG.0000278567.37925.4e

[B10] Hefni M El-Toukhy T Bhaumik J Katsimanis E Sacrospinous cervicocolpopexy with uterine conservation for uterovaginal prolapse in elderly women: an evolving concept Am J Obstet Gynecol 2003 188 645 50 10.1067/mob.2003.75 12634635

[B11] Morgan DM Rogers MA Huebner M Wei JT Delancy JO Heterogeneity in anatomic outcome of sacrospinous ligament fixation for prolapse: a systematic review Obstet Gynecol 2007 109 1424 33 10.1097/01.AOG.0000264066.89094.21 17540817

[B12] Diwan A Rardin CR Kohli N Uterine preservation during surgery for uterovaginal prolapse: a review Int Urogynecol J Pelvic Floor Dysfunct 2004 15 286 92 15517676 10.1007/s00192-004-1166-4

[B13] Dietz V de Jong J Huisman M Schraffordt Koops S Heintz P van der Vaart CH The effectiveness of the sacrospinous hysteropexy for the primary treatment of uterovaginal prolapse Int Urogynecol J Pelvic Floor Dysfunct 2007 18 1271 6 10.1007/s00192-007-0336-6 17384894

[B14] Dietz V Huisman M de Jong J Heintz P van der Vaart CH Functional outcome after sacrospinous hysteropexy for uterine descensus Int Urogynecol J Pelvic Floor Dysfunct 2008 19 747 52 10.1007/s00192-007-0520-8 18297228 PMC2335287

[B15] Maher CF Cary MP Slack MC Murray CJ Milligan M Schluter P Uterine preservation or hysterectomy at sacrospinous colpopexy for uterovaginal prolapse? Int Urogynecol J Pelvic Floor Dysfunct 2001 12 381 385 10.1007/s001920170017 11795641

[B16] Brummen HJ van de Pol G Aalders CIM Heintz APM van der Vaart Sacrospinous hysteropexy compared to vaginal hysterectomy as primary surgical treatment for a descensus uteri: effect on urinary symptoms Int Urogynecol J Pelvic Floor Dysfunct 2003 14 350 355 10.1007/s00192-003-1084-x 14618315

[B17] Hefni MA El-Toukhy TA Long-term outcome of vaginal sacrospinous colpopexy for marked uterovaginal and vault prolapse Eur J Obstet Gynecol Reprod Biol 2006 127 257 263 10.1016/j.ejogrb.2005.11.028 16377061

[B18] Dietz V van der Vaart CH van der Graaf Y Heintz P Schraffordt Koops SE One-year follow-up after sacrospinous hysteropexy and vaginal hysterectomy for uterine descent: a randomized study Int Urogynecol J Pelvic Floor Dysfunct 2010 21 209 16 10.1007/s00192-009-1014-7 19834635 PMC2808513

[B19] Bump RC Mattiasson A Bo K Brubaker LP DeLancey JO Klarskov P Shull BL Smith AR The standardization of terminology of female pelvic organ prolapse and pelvic floor dysfunction Am J Obstet Gynecol 1996 175 10 7 10.1016/S0002-9378(96)70243-0 8694033

[B20] Hall AF Theofrastous JP Cundiff GW Harris RL Hamilton LF Swift SE Bump RC Interobserver and intraobserver reliability of the proposed International Continence Society, Society of Gynecologic Surgeons, and American Urogynecologic Society pelvic organ prolapse classification system Am J Obstet Gynecol 1996 175 1467 70 10.1016/S0002-9378(96)70091-1 8987926

[B21] Ware JE Kosinski M Keller SD SF-36 physical and mental component summary measures-a users' manual 1994 Boston: New England Medical Center, The Health Institute

[B22] van der Zee KI Sanderman R Het meten van de gezondheidstoestand met de Rand-36, een handleiding 1993 Groningen: Noordelijk centrum voor gezondheidsvraagstukken

[B23] van der Vaart CH de Leeuw JR Roovers JP Heintz AP Measuring health-related quality of life in women with urogenital dysfunction: the urogenital distress inventory and incontinence impact questionnaire revisited Neurourol Urodyn 2003 22 97 104 10.1002/nau.10038 12579625

[B24] Roovers JP van der Bom JG van der Vaart CH Heintz AP Prediction of findings at defecography in patients with genital prolapse BJOG 2005 112 1547 1553 10.1111/j.1471-0528.2005.00734.x 16225577

[B25] Dolan P Modeling valuations for EuroQol health states Med Care 1997 35 1095 1108 10.1097/00005650-199711000-00002 9366889

[B26] Lamers LM Stalmeier PF McDonnell J Krabbe PF van Busschbach JJ Measuring the quality of life in economic evaluations: the Dutch EQ-5D tariff NTVG 2005 149 1574 8 16038162

[B27] Espuña Pons M Sexual health in women with pelvic floor disorders: measuring the sexual activity and function with questionnaires - a summary Int Urogynecol J Pelvic Floor Dysfunct 2009 20 S65 S71 19440785 10.1007/s00192-009-0828-7

[B28] Schweitzer KJ de Jong M Milani AL Prolaps en seks: hoe meten we de relatie? NTOG 2008 121 79 82

[B29] Vroege JA De vragenlijst voor het signaleren van seksuele dysfuncties (VSD). Bruikbaarheid in de klinische praktijk 2003 Delft

[B30] Kluivers KB Hendriks JC Mol BW Bongers MY Vierhout ME Brölmann HA de Vet HC Clinimetric properties of 3 instruments measuring postoperative recovery in a gynecologic surgical population Surgery 2008 144 12 21 10.1016/j.surg.2008.03.027 18571580

